# Hemorrhage segmentation in mobile-phone retinal images using multiregion contrast enhancement and iterative NICK thresholding region growing

**DOI:** 10.1038/s41598-022-26073-6

**Published:** 2022-12-13

**Authors:** Patsaphon Chandhakanond, Pakinee Aimmanee

**Affiliations:** School of Information, Computer, and Communication Technology, Sirindhorn International Institute of Technology, Thammasat University, 131 Moo 5, Tivanont Rd, Bangkadi, Meung, Patumthani, 12000 Thailand

**Keywords:** Computational biology and bioinformatics, Image processing

## Abstract

Hemorrhage segmentation in retinal images is challenging because the sizes and shapes vary for each hemorrhage, the intensity is close to the blood vessels and macula, and the intensity is often nonuniform, especially for large hemorrhages. Hemorrhage segmentation in mobile-phone retinal images is even more challenging because mobile-phone retinal images usually have poorer contrast, more shadows, and uneven illumination compared to those obtained from the table-top ophthalmoscope. In this work, the proposed *KMMRC-INRG* method enhances the hemorrhage segmentation performance with nonuniform intensity in poor lighting conditions on mobile-phone images. It improves the uneven illumination of mobile-phone retinal images using a proposed method, *K-mean multiregion contrast enhancement *(*KMMRC*). It also enhances the boundary segmentation of the hemorrhage blobs using a novel *iterative NICK thresholding region growing* (*INRG*) method before applying an SVM classifier based on hue, saturation, and brightness features. This approach can achieve as high as 80.18%, 91.26%, 85.36%, and 80.08% for recall, precision, F1-measure, and IoU, respectively. The F1-measure score improves up to 19.02% compared to a state-of-the-art method DT-HSVE tested on the same full dataset and as much as 58.88% when considering only images with large-size hemorrhages.

## Introduction

A mobile phone with a unique portable retinal lens can conveniently produce many retinal images. Its portability and economic cost attract many health care organizations to use it to prescreen ophthalmic diseases such as glaucoma and diabetic retinopathy (DR) for patients on a large scale^1^. However, mobile-phone retinal images have undesirable characteristics, such as blurry edges, nonuniform illumination, shadowy background, and low contrast.

Hemorrhages are abnormal bleeding from retinal blood vessels. They are one of the prime indicators of DR. The shapes and shades of hemorrhages vary. Common shapes are dome, semilunar, crescentic, plaque, splinter, flame, lozenge, pool, and dot^1,3^. Some may appear as irregular geographic shapes. The colors of the hemorrhages are determined by the levels of the affected retina, leakage amounts, and ages^2–4^. Moreover, there can be more than one shade in a single hemorrhage. Hemorrhage detection is generally difficult not only because of wide variations in shapes and shades but also because of the high similarity to blood vessels and shadows in the image. The reviews of work related to hemorrhage detection and segmentation in retinal images are summarized in Table 1. Most work on hemorrhage segmentation completely neglects the fact that a single hemorrhage can have nonuniform intensity, especially large hemorrhages. When a specific intensity range is used for hemorrhage segmentation, incomplete regions are usually segmented, resulting in a low recall value.

**Table 1:** A table summarizes the hemorrhage detection and segmentation techniques used in the literature. AUC is area under the ROC curve.

Authors	Methods/techniques	Dataset and size	Evaluation	Performance		Advantages	Drawbacks limitations
				Metrics	Value (%)		
Murugan^2^ (2019)	The motion patterns generation algorithm to detect the hemorrhages	1200 retinal images of a Messidor public dataset	Detection	Sensitivity Specificity	97.00 98.00	This method can reduce the dimensional space based on image resolutions, thereby speeding up hemorrhage detection.	The algorithm requires high computational time. On a PC, it requires, on average, 1.5 min for each image.
Manjaramkar et al.^3^ (2017)	The connected component clustering method based on maximally stable extremal regions (MSER) for detecting many occurrences of hemorrhages with different shapes and sizes	> 65 images from STARE and Messidor public datasets	Detection	Sensitivity Specificity	96.45 94.89	This method distinguishes between hemorrhages of any size, shape, or appearance, including massive, isolated, and vessel-connected hemorrhages. Hemorrhages can be detached or vessel-related.	The proposed method does not work well when the image has low contrast and the image has no hemorrhage.
Karkuzhali et al.^5^ (2018)	The K-means algorithm and a morphological approach to efficiently segment all types of hemorrhages. K-means clustering classifies a given dataset into a fixed number of clusters based on pixel or group pixel similarity. It classifies each pixel in a group based on the centroid's mean distance from the pixel. This algorithm clusters the data in an iteration process by calculating the mean intensity for each group	380 images from a STARE public dataset	Segmentation	Sensitivity Specificity Accuracy	97.00 92.00 93.00	The method is considered a rapid automatic detection method for efficiently segmenting all types of hemorrhages.	The proposed method only works well when the size of the Lesion is distinct from the hemorrhage size.
Godlin et al.^6^ (2018)	The modified RG (MRG), a region growing (RG) with a threshold established by gray wolf optimization (GWO)	50 fundus images from Messidor public dataset	Segmentation	Accuracy	92.56	The optimal threshold value is predicted for improved segmentation, enhancing the accuracy of segmented image optimization techniques. Gray wolf optimization (GWO) is used to improve accuracy via this optimal threshold process.	The proposed method does not work well with the bright lesion.
Tang et al.^7^ (2013)	The splats (image segments) to segment hemorrhages. The image is divided into nonoverlapping splats of similar intensity that cover the entire image. Each splat comprises pixels that share a similar color and spatial location	1,200 fundus images from the Messidor public dataset	Segmentation	AUC	96.00	Regardless of their various appearance, texture, or size, splat-based feature classification could model the shapes of various lesions with efficiency.	Susceptible to the lesion with brightness and size similar to hemorrhages. This leads to classification errors.
Adem et al.^8^ (2018)	The iterative thresholding method based on the firefly algorithm (FFA) and particle swarm optimization algorithm (PSOA). In the iterative thresholding step, the number of hemorrhagic regios and the pixel counts in these regions are determined using an iterative thresholding technique that generates different thresholding values with the FFA/PSOA	100 images from a local dataset	Detection	Sensitivity Specificity Accuracy	96.70 91.40 94.10	It obtains high evaluation scores.	It easily falls into a local optimum in high-dimensional space and has a low convergence rate in the iterative process.
Gupta et al.^9^ (2014)	The multiresolution morphological processing for the perceptual clustering of hemorrhagic candidates of various sizes, shapes, and textures	191 images from a local dataset	Detection	Sensitivity	>82.00	This method allows the perceptual grouping of hemorrhagic candidates with various sizes, shapes, and textures. It achieves an accurate segmentation of true hemorrhages, including those attached to vessels.	The sensitivity is higher for candidates with larger sizes.
Ruennak et al.^10^ (2021)	The DT-HSVE, an Adaptive thresholding method, and a decision tree classifies them based on hue, saturation, brightness, and edge sharpness features	134 images taken by a mobile phone from a local dataset	Segmentation	Recall Precision	62.26 71.01	Simple algorithms, yield acceptable results.	Low hemorrhage segmentation rate Time to process an image is still high (90 secs/image).
Maqsood et al.^11^ (2021)	The 3D convolutional neural network (CNN) architecture for hemorrhage detection, and transfer learning-based feature extraction for retraining the modified VGG19-based CNN model, MRCEV algorithm for feature selection, and ELM classifier for hemorrhage detection	1509 images from several public datasets: HRF, DRIVE, STARE, Messidor, DIARETDB0, and DIARETDB1 databases	Detection	Accuracy	97.71	It yields high accuracy	The execution time range for hemorrhage detection is 15.46–17.54 s, which is quite lengthy
Tan et al.^12^ (2017)	The convolutional neural network (CNN) with 10 layers to automatic segregation and differentiation of exudates, hemorrhages, and microaneurysms	149 images from public dataset CLEOPATRA	Detection	Sensitivity	62.57	Automatically and simultaneously, the algorithm can segment and differentiate exudates, hemorrhages, and microaneurysms.	The sensitivities of the hemorrhage detection rate are quite low.
Srivastava et al ^13^ (2017)	The multiple kernel education (MKL). The radial basis function (RBF) kernel is used as the kernel in this study. The input image was divided into patches to detect lesions of various sizes. Different grid sizes were used to subdivide the image, and the information from each grid size was combined using 295 MKL	143 images from public datasets: DIARETDB13 and MESSIDOR4	Detection	AUC	92.00	Using MKL was found to improve performance in comparison to using a single grid size.	Using different grid sizes has the drawback of requiring intensive computations for larger grid sizes.
Dai et al.^14^ (2021)	The deep-learning systemDeepDR. Three deep-learning subnetworks comprised the DeepDR system: image quality assessment subnetwork, lesion-aware subnetwork, and DR grading subnetwork	409,458 images of the local dataset	Detection	AUC	96.70	In DR grading, the DeepDR system achieved high sensitivity and specificity. Instead of generating a DR grading, it provides visual cues that assist users in identifying the presence and location of various lesion types.	DeepDR requires additional external validation in multiethnic and multicenter cohorts to confirm the robustness of lesion detection and DR grading.
Asiri et al.^15^ (2021)	The deep-learning-based unified framework for red lesion detection technique. To simultaneously detect small and large red lesions, it used two streams of faster region-based convolutional neural network (RCNN). Each stream utilizes the VGG-16 model with ROI pooling, regression, and classification layers	Public datasets: DiaretDB1-MA, DiaretDB1-HM, e-ophtha and ROCh	Detection	AUC	90.52–98.64	High accuracy	The proposed method cannot detect small or thin hemorrhages well.
Khojasteh et al.^16^ (2018)	The convolution neural networks (CNN) for detection of exudates, hemorrhages, and microaneurysms. Then calculated the probability that each pixel contributed to one of four classes: exudate, hemorrhage, microaneurysm, and background (no pathologic sign)	284 images from a DIARETDB1 public dataset and 209 images from an e-Ophtha public dataset	Segmentation	Precision Sensitivity	86.60–97.00 84.00–96.00	Using score values obtained from the softmax layer as opposed to the network's binary output. This results in the generation of the probability map of the locations of the pathological signs on the image, which, when appropriately post processed, reduces the error rate in the size of the signs.	The method cannot differentiate between hemorrhages and microaneurysms. The database of 284 images contained very few hemorrhagic images.

The hemorrhage characteristics and the poor image quality produced by a mobile phone make hemorrhage segmentation even more challenging. Light exposure and illumination commonly found in mobile phone retinal images make contrast enhancement ineffective. The shadows directly affect hemorrhage segmentation performance because they are often incorrectly segmented as a hemorrhage, resulting in low precision. Images with both shadows, light exposure, and illumination considered poor quality are generally more difficult to detect hemorrhages than good-quality images.

This work improves hemorrhage segmentation performance, especially for large hemorrhages with nonuniform intensity, in a poor-quality retinal dataset from a mobile phone.

## Objectives and contributions

Improving the hemorrhage segmentation performance for a mobile phone retinal dataset is the main objective of this work. A novel method *KMMRC-INRG* is proposed. The method considers the problems of uneven background illumination characteristic of a mobile phone retinal dataset and the nonuniform intensity of the hemorrhages. It comprises two new subalgorithms: *K-mean multiregion contrast enhancement *(*KMMRC*) and *iterative NICK thresholding region growing *(*INRG*)*.* The KMMRC algorithm overcomes the intense illumination and poor contrast in mobile phone images. The *INRC* algorithm overcomes multishading hemorrhages.

The findings of this work add a new theoretical contribution to the existing knowledge in terms of techniques. The improvement of hemorrhage segmentation performance is a practical contribution. It can directly help improve the DR prescreening and severity grading performances. The proposed algorithms can be used in other applications that involve multishading objects such as an optic disk or skin cancers.

## Methodology

**Figure 1:**
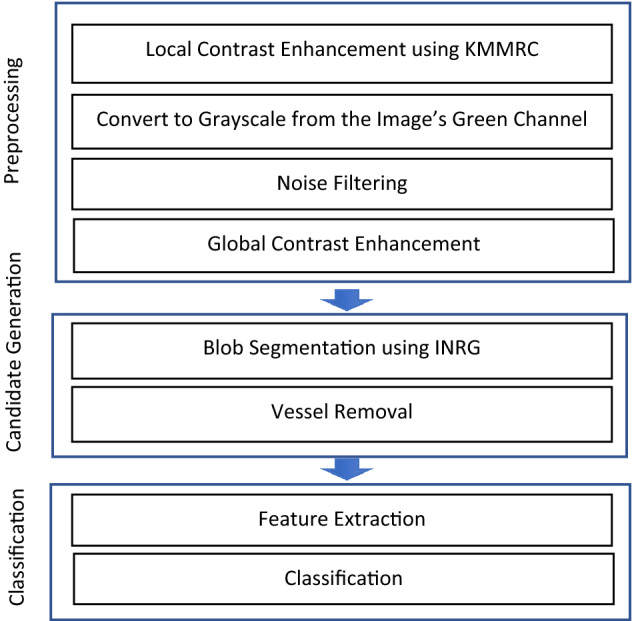
Main processes of the KMMRC-INRG method.

Figure 1 shows the framework of KMMRC-INRG. It comprises three main steps: preprocessing, candidate generation, and classification. KMMRC and INRG are novel algorithms proposed in this work. The details of the algorithms are provided in the later sections. The following describes the details of each step.

### A. Preprocessing

As the green channel of the image can help detect better dark components such as hemorrhages^17^, we convert the image’s green channel to grayscale and use it as an input. Preprocessing comprises two main tasks. First are the contrast enhancements, which are performed locally and globally. The second is noise removal.

Local contrast enhancement is first applied to improve local contrast because the background has areas with different lighting conditions, such as illumination and shadows. We proposed a *K-means multiregion contrast enhancement* (KMMRC) algorithm to improve the uneven lighting background. The *K*-*means* algorithm^18^ is used to divide the background into *K* regions based on intensity. A linear edge enhancement algorithm then enhances the contrast of each region. The pseudoalgorithm is provided in Algorithm 1.
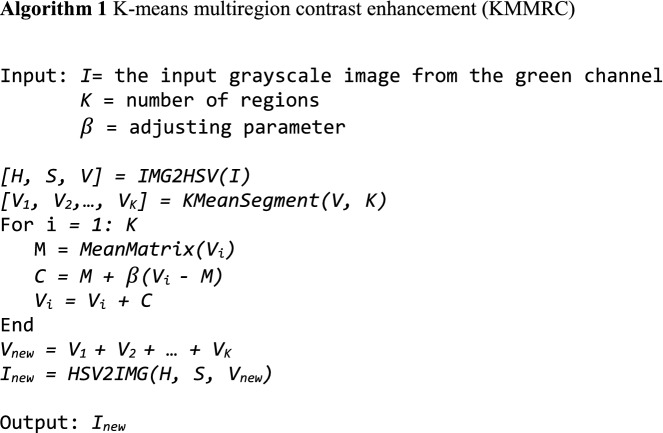


The functions used in the algorithm are defined as follows.

*IMG2HSV(I)* takes the input image *I* and extracts and returns the hue (*H*), saturation (*S*), and brightness (*V*) matrices of *I*.

*KMeanSegment(V, K)* takes a brightness matrix *V* and an integer *K* as inputs. Based on brightness values, it divides *V* into *K* submatrices *V*_*1*_*, V*_*2*_*, …, V*_*K*_ of which *V*_*1*_ + *V*_*2*_ + *…* + *V*_*K*_ = *V*.

*MeanMatrix(V)* takes a brightness matrix *V* as an input. It finds the average values in a matrix *V* and returns a matrix of the same dimension as *V,* of which all elements hold the value of the computed average.

*HSV2IMG(H, S, V)* takes the matrices hue (*H*), saturation (*S*), and brightness (*V*) as inputs. It constructs and returns an image from the input matrices *H, S,* and *V.*

Our empirical observations show that *K* = 5 and $\beta =1.5$ give the best performance. We then apply contrast-limited adaptive histogram equalization (CLAHE)^19^ to the image to smooth the false edges resulting from local contrast enhancement. To remove the noise and smoothen the image, we apply average filtering^20^ to the resultant image.

### B. Candidate generation

We used the contrast-enhanced images from the previous step as input. In this step, the hemorrhage candidates from the contrast-enhanced images are created. To obtain the hemorrhage candidates, we performed blob segmentation and vessel removal. The blob segmentation is performed by using the proposed iterative NICK thresholding region growing (INRG) algorithm. The pseudoalgorithm of INRG is shown in Algorithm 2.
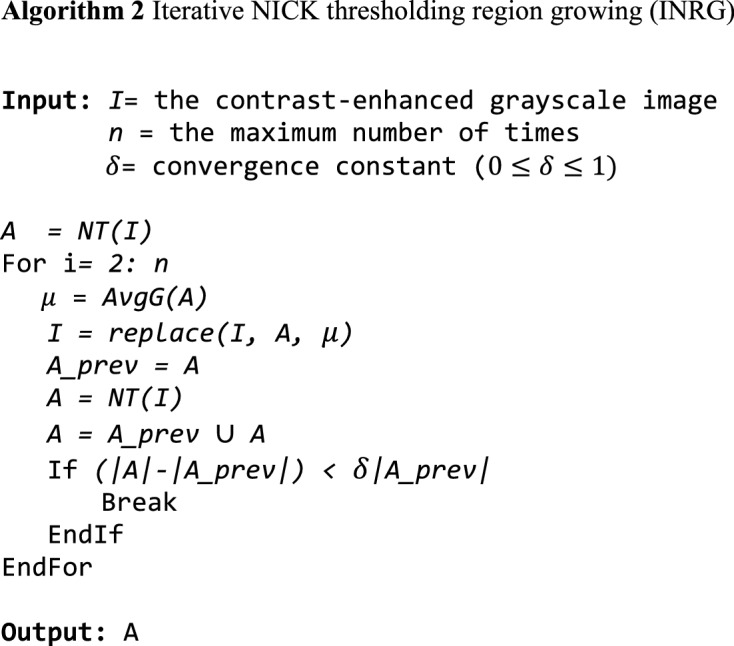


The INRG algorithm uses the following functions.

*NT(I)* takes an image *I* as an input and returns a set of *(x, y)* coordinates of points that pass the NICK threshold in Eq. (1).

1 $$\tau (x,y)=avg\left(x,y\right)+\kappa \sqrt{\frac{(\sum \left({V}^{2}\left(x,y\right)\right) - av{g}^{2}\left(x,y\right))}{N}},$$

where $avg\left(x,y\right)$ is a local average at $\left(x, y\right)$, $V\left(x,y\right)$ is the intensity at $\left(x, y\right)$, *N* is the number of points in the area, and $\kappa$ is a parameter in the range [−0.2, −0.1]. From the experiment, we find that $\kappa$ = −0.2 and N = 9025 yield the best result.

*replace(I, A, c)* takes an image *I*, a set of points *A*, and a value *c* as inputs. It replaces the values of points *A* in *I* with c.

*AvgG(I, A)* takes an image *I* and a set of points *A* as inputs and returns the average green values of all points in *A*.

Remark $\cup$ is a union operator. Our empirical observations show that *n* = 3 and $\delta =0.1$ give the best performance.

**Figure 2:**
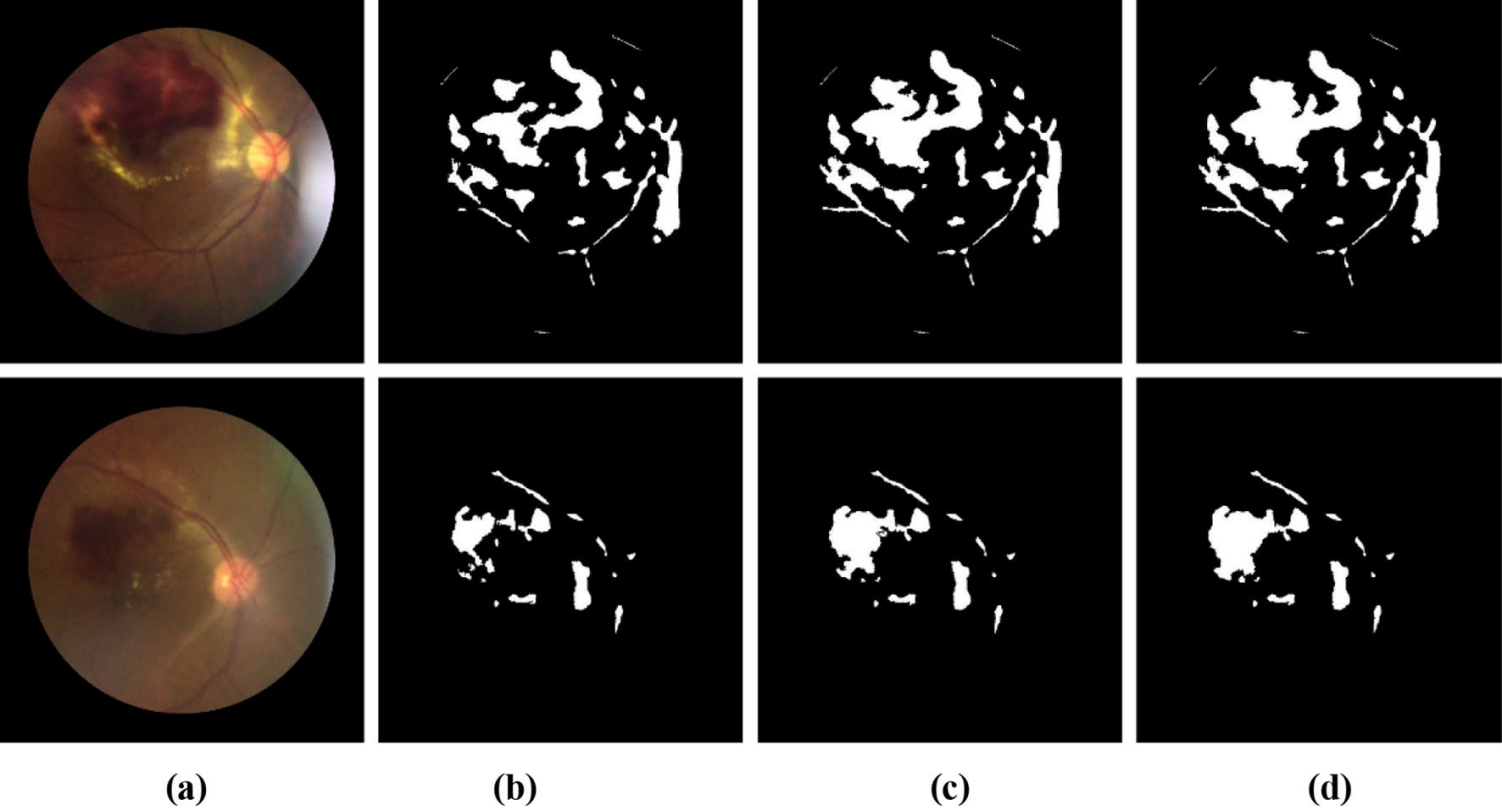
Images of blob regions at different iterations of INRG. The original images (**a**) and regions obtained from the INRG algorithm from the first iteration until it converges (**b–d**).

The algorithm first calculates the initial regions of the candidate blobs by searching for pixels that are salient compared to their local backgrounds using NICK thresholding (NT)^21,22^. To better extract a region with nonuniform intensity, the algorithm expands the region by replacing the intensity in the regions with its average. Then, the algorithm reapplies NICK thresholding. It repeats until the region’s growth rate is less than a convergence constant. The program repeats at most *n-1* times to ensure a complete exit. Figure 2 shows the areas of hemorrhage candidates using the INRG algorithm at different iterations until it converges.

Long and thin blobs are usually vessels. The algorithm detects these blobs by considering the axis length ratio of the fitted ellipse's major and minor axes. From an empirical experiment, the ratio of 6.4 gives the optimal solution; it is assumed to be a blood vessel and is removed.

### C. Feature extraction and classification

The hue-saturation-value (HSV) color space is used in the feature extraction process, as hemorrhages are usually rich in color, have low saturation, and have low brightness. Additionally, HSV is more resistant to external lighting than RGB. The *H*, *S*, and *V* values are used as features extracted from each candidate.

**Figure 3:**
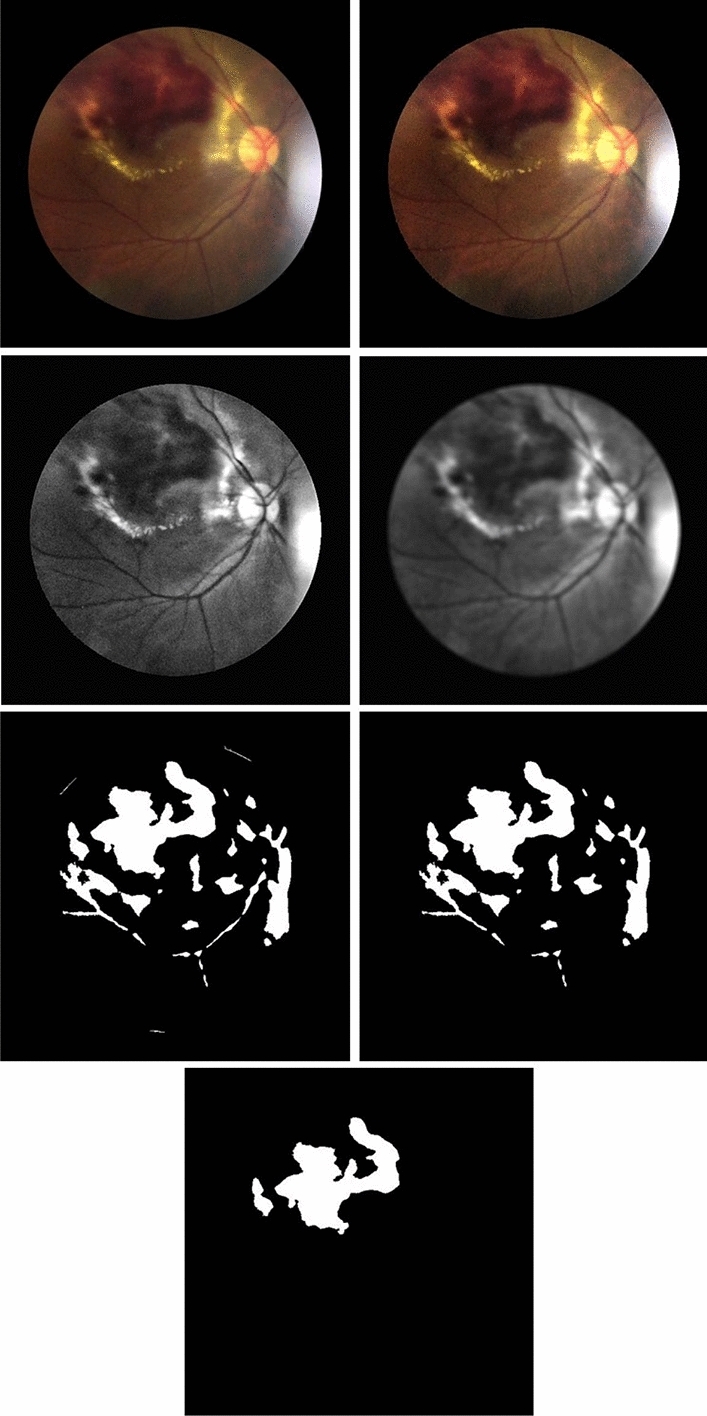
Illustration of overall processes to obtain hemorrhage: original image (top left), after applying KMMRC to adjust local contrast (top right), after conversion to a grayscale of the green channel and global contrast enhancement using CLAHE (second row-left), after noise filtering using average filter (second row-right), after region segmentation using INRG algorithm (third row-left), after vessel removal (third row-right), and after classification (bottom).

Blob’s feature data and correct class answers (hemorrhagic and nonhemorrhagic) are trained and tested using fivefold cross-validation. Figure 3 depicts all processes from the beginning until hemorrhages are obtained.

## Dataset and evaluation

We used a retrospective mobile-phone retinal dataset^10^ comprising 100 images with a 50–50 ratio of hemorrhagic and nonhemorrhagic images taken by an iPhone 6s with a Volk iNview retinal lens. The data were collected from Thammasat Chalermprakiat Hospital in Thailand in 2019. The images were of type jpg and were all of dimension 598 × 597. Generally, the images had a narrower field of view than retinal images produced from standard ophthalmoscopes. Statistically, there were 32 images with blurry edges, 60 images with light explosive areas, 81 images with shadows, 50 images with uneven illumination, and 8 images with large hemorrhages with nonhomogeneous shades. There were 31 hemorrhagic images in the collection. We evaluate the performance of KMMRC-INRG in hemorrhage segmentation using standard recall, precision, F1-measure and intersection over union (IoU). The formulas of these evaluations are as follows.

$$\mathrm{Recall }=\frac{TP+TN}{TP+TN+FP+FN},$$

$$\mathrm{Precision}=\frac{TP}{TP+FP},$$

$$\mathrm{F}1-\mathrm{measure }=2 \frac{precision \times recall}{precision + recall},$$

$$\mathrm{IoU }=\frac{TP}{TP+FN+FP}$$

where TP, TN, FP, and FN are the number of blobs that are true positive, true negative, false positive, and false negative, respectively.

For image classification**,** we used a full set of 100 images from the mobile retinal dataset. We used the KMMRC-INRG algorithm to detect hemorrhages. When a hemorrhage was detected in an image, we considered the image positive; otherwise, it was considered negative. We evaluated the performance of the algorithm for classifying images using true positives, true negatives, false positives, and false negatives. The confusion matrix was analyzed and interpreted in terms of sensitivity, specificity, positive predictive value (PPV), and accuracy^10^. The formulas for these evaluations are as follows.

$$\mathrm{Sensitivity}=\frac{T{P}_{I}}{T{P}_{I}+F{N}_{I}},$$

$$\mathrm{Specificity}=\frac{T{N}_{I}}{T{N}_{I}+F{P}_{I}}$$

$$\mathrm{PPV}=\frac{T{P}_{I}}{T{P}_{I}+F{P}_{I}}$$

$$\mathrm{Accuracy }=\frac{T{P}_{I}+T{N}_{I}}{T{P}_{I}+F{P}_{I}+T{N}_{I}+F{N}_{I}}$$

where *TP*_*I*_, *TN*_*I*_*, FP*_*I*_*,* and *FN*_*I*_ are the number of images that are true positive, true negative, false positive, and false negative, respectively.

## Results

**Figure 4:**
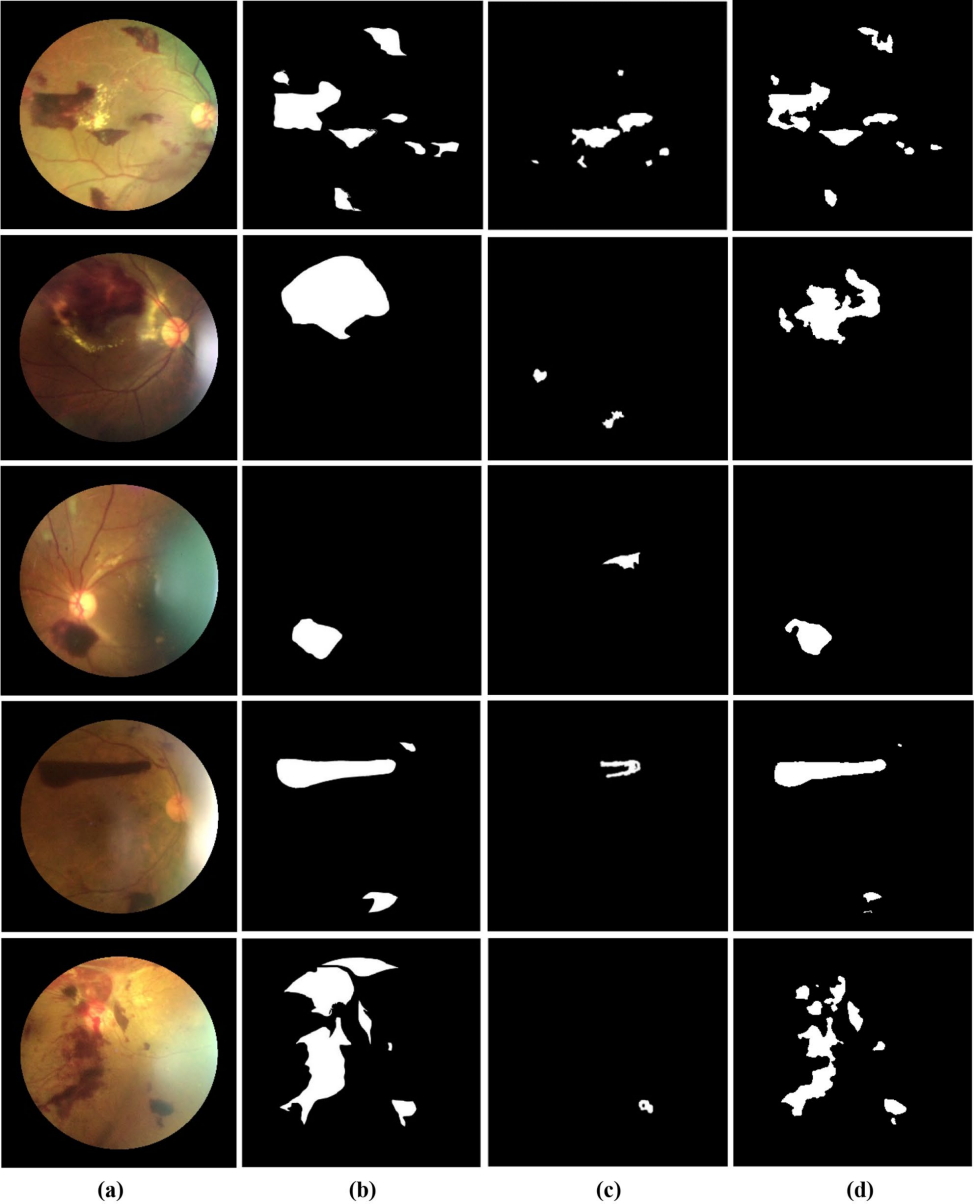
Hemorrhage segmentation comparison: (**a**) original images, (**b**) ground truths, (**c**) DT-HSVE^10^, and (**d**) our proposed method KMMRC-INRG (**d**).

The results of hemorrhage segmentation of our proposed method were compared against DT-HSVE^10^ and three other variants of the proposed method: XKMMRC-NT, XKMMRC-INRG, and KMMRC-NT. The XKMMRC-NT performs all KMMRC-INRG processes except the KMMRC, and NT was used instead of INRG. This variant is the base model when the two proposed algorithms are turned off. The XKMMRC-INRG performs all KMMRC-INRG processes except the KMMRC step. KMMRC-NT performs all KMMRC-INRG processes except that NT is used instead of INRG. Note that NT is the same as INRG with *k* = 1 (no region growing step). All models, including the comparative method DT-HSVE, are tested on the same dataset. The qualitative results of selected examples of images tested by KMMRC-INRG are shown in Fig. 4. The numerical results are shown in Table 2.

**Table 2:** Hemorrhage segmentation performance comparisons of KMMRC-INRG against comparative methods in the percentage of all images.

Methods	Recall	Precision	F1-measure	IOU
DT-HSVE^10^	62.26	71.01	66.34	63.12
XKMMRC-NT	77.14	**93.93**	84.71	79.22
XKMMRC-INRG	78.88	91.75	84.83	78.94
KMMRC-NT	79.39	92.37	**85.39**	**80.27**
KMMRC-INRG (proposed)	**80.18**	91.26	85.36	80.08

The highest values are bold.

KMMRC-INRG outperforms all comparative methods in terms of recall. The recall, precision, F1-measure, and IoU of KMMRC-INRG are higher than those of DT-HSVE^10^ by 17.92%, 20.24%, 19.02% and 16.96%, respectively. The performances of KMMRC-INRG and its three variants (XKMMRC-NT, XKMMRC-INRG, and KMMRC-NT) are not significantly different. It is worth noting that DT-HSVE outperforms adaptive thresholding, region growing, and the watershed method on the same dataset and ground truth. As KMMRC-INRG outperforms DT-HSVE, it also outperforms all comparative methods of DT-HSVE by implication.

As KMMRC-INRG is designed to improve the detection of large-size hemorrhages, which usually have a nonhomogenous intensity, the performance of KMMRC-INRG depends on the number of images with these characteristics. The higher the value is, the better the improvement. Thus, we consider images with large hemorrhages from the same dataset to see the performance of the proposed method on the targeted images. Table 3 shows the segmentation performance on eight images with large hemorrhages. A hemorrhage is large when the ratio of the hemorrhagic area to the circular area of the retina is greater than 3.5%.

**Table 3:** Hemorrhage segmentation performance comparisons of KMMRC-INRG against comparative methods in the percentage of eight images with large hemorrhages.

Methods	Recall	Precision	F1-measure	IOU
DT-HSVE^10^	12.30	25.23	16.53	15.23
XKMMRC-NT	52.03	97.06	67.89	67.42
XKMMRC-INRG	59.48	97.03	73.75	71.00
KMMRC-NT	54.32	**97.18**	69.69	68.29
KMMRC-INRG (proposed)	**62.02**	96.18	**75.41**	**72.44**

The highest values are bold.

The results of performance comparisons on images with large hemorrhages show that KMMRC-INRG significantly outperforms DT-HSVE. The absolute improvement is as high as 58.88%. KMMRC-INRG improves the recall, F1-measure, and IoU scores of the base model XKMMRC-NT by 9.99%, 7.52%, and 5.02%, respectively. This implies that the two proposed algorithms KMMRC and INRC help considerably improve segmentation performance. KMMRC-INRG’s F1-measure and IoU scores are the highest compared to its three variants. The improvement of KMMRC-INRG’s recall over the XKMMRC-INRG of 2.54% implies the performance of using just only KMMRC. Additionally, the KMMRC-INRG’s recall performance is greater than KMMRC-NT by 7.70%. We can conclude from the improvement of KMMRC-INRG over KMMRC-NT that applying NICK thresholding (NT) in more than one round (INRG) can better segment a hemorrhagic region.

The next section shows the image classification results using KMMRC-INRG. The results in Table 4 show that the proposed work classifies the hemorrhagic images from nonhemorrhagic images very well. The accuracy obtained is as high as 89.00%.

**Table 4:** Confusion matrix of image classification.

		Predicted class	
		Has-hemorrhage	Has-no hemorrhage
True class	Has-hemorrhage	44	6
	Has-no hemorrhage	5	45
**Image classification evaluation**			
Sensitivity		88.00	
Specificity		89.80	
PPV		83.72	
Accuracy		89.00	

**Figure 5:**
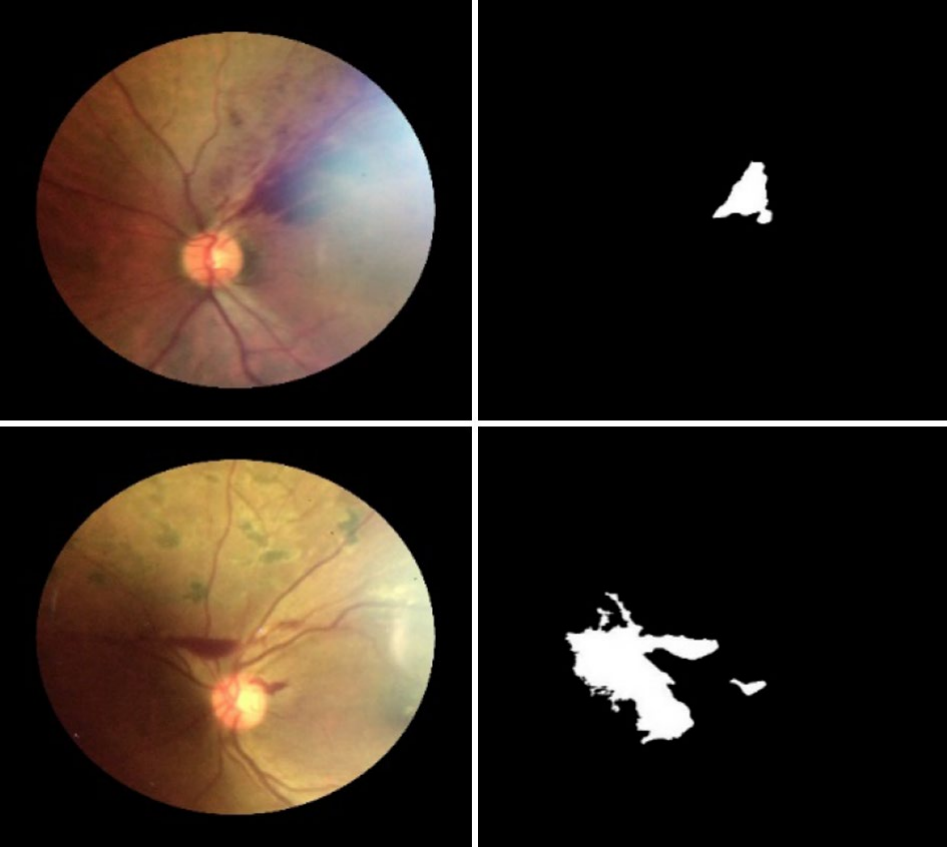
Examples of an undersegment case (top) and an oversegment case (bottom). The original image and the segmented result from KMMRC-INRG are shown on the left and the right, respectively.

We look at cases that obtained low F1-measure scores to analyze the causes. The reasons are as follows. First, they appear in a light explosive area. The second is because a hemorrhage is within a dark shadow environment. Figure 5 shows two examples of such cases. When a hemorrhage appears in the light explosive area, the algorithm does not repeat because the brightness difference is low. Consequently, the area is undersegmented. The opposite scenario occurs when a hemorrhage is in a dark environment. In this case, the average intensity of each round is low and causes the INRG algorithm to repeat too many times, resulting in oversegmentation. Improving contrast more efficiently is our future work.

## Conclusion

The KMMRC-INRG method is proposed in this work. It uses novel K-mean multiregion clustering (KMMRC) to improve the uneven background of mobile-phone retinal datasets. It utilizes a newly proposed method INRG that helps improve the segmentation of a multishade hemorrhage. The SVM is used to classify the candidate hemorrhage blobs based on hue, saturation, and brightness features (HSVs). The KMMRC-INRG method can classify hemorrhagic images from nonhemorrhagic images with up to 89.00% accuracy. It can generally segment hemorrhages with an average F1-measure score of 85.36% on a mobile-phone retinal dataset, which is 19.02% higher than DT-HSVE, the state-of-the-art method. The improvement is even more significant in images with large hemorrhages, which is as much as 58.88%.

## Data Availability

The datasets generated and/or analyzed during the current study are available in Google drive at the following link. https://drive.google.com/drive/u/0/folders/1oK2fHPHxtiPDVaPa3A1IuquapqC_KCAd.
